# COVID-19 in Space: Possible Health Risks and Preparedness Guidelines

**DOI:** 10.3390/pathogens15050498

**Published:** 2026-05-06

**Authors:** Ishan Vashishat, Sanghyun Eddie Han, Barnabe D. Assogba

**Affiliations:** 1 Faculty of Science, Department of Biology & Health Science, Kwantlen Polytechnic University, Surrey, BC V3W 2M8, Canada; ishan.vashishat@student.kpu.ca (I.V.); eddie.han1@kpu.ca (S.E.H.); 2 Department of Biology, Douglas College, New Westminster, BC V3M 5Z5, Canada

**Keywords:** COVID-19, microgravity, space, airborne infectious disease, systematic review

## Abstract

Background: The COVID-19 pandemic resulted in over 705 million infections and 7 million deaths, underscoring the importance of understanding disease behavior across diverse environments. As NASA, SpaceX, and ISRO prepare for more frequent missions, managing health risks for astronauts and space tourists is essential. Objective: This study reviews the literature on airborne infections in space, identifies research gaps, and establishes preparedness strategies for potential COVID-19 outbreaks during space missions. Methods: A systematic literature review was conducted to identify studies examining airborne infectious diseases in space. To compare these findings with Earth-based data, pathogen safety data sheets were used. A separate systematic review was conducted to explore similarities between COVID-19 and the identified airborne infectious diseases. A comparative approach was used to predict COVID-19’s potential behavior in microgravity. Existing guidelines for managing airborne diseases in space and on Earth were reviewed and compared to develop a set of preparedness recommendations for COVID-19 in space. Results: Nine airborne infectious diseases occurring in space were identified. Six tentative effects of COVID-19 in a microgravity environment were theorized in this study. We propose recommendations to improve current space travel health guidelines and address the identified risks. Conclusions: The results of this study will change the course of human space exploration by assisting in the protection of space travelers and guiding the development of new protocols that include comprehensive safety features.

## 1. Introduction

Human space travel, which began in 1961, has seen 570 people journey beyond Earth’s atmosphere and return by 2021, with some individuals undertaking as many as seven missions during their careers [1]. The number of space travelers continues to grow, with 25 individuals living and working on the International Space Station by 2024, as reported by NASA [2]. However, a significant decline in space travel occurred in 2020 and 2021, with only 12 and 7 individuals traveling to space during those years, respectively, owing to disruptions caused by COVID-19 [1]. The COVID-19 pandemic has led to global lockdowns and a significant loss of human life, severely affecting space operations and healthcare systems on Earth. If a similar outbreak occurs during a space mission, it could jeopardize mission success. However, the effects of COVID-19 in extreme microgravity environments remain unknown.

### 1.1. COVID-19 as a Viral Vector

The year 2020 marked the emergence of COVID-19, an airborne infectious disease that rapidly spread across more than 200 countries. COVID-19 has reportedly infected over 776 million people, causing widespread social and economic disruption [3,4]. The name “COVID-19,” which stands for Coronavirus Disease 2019, reflects its discovery in 2019. The disease is caused by the severe acute respiratory syndrome coronavirus type 2 (SARS-CoV-2), a single-stranded RNA virus [3,5]. SARS-CoV-2 transmission occurs through respiratory droplets and aerosols generated during normal breathing, coughing, sneezing, or speaking, making it readily transmissible in confined environments [3]. Once inside the human body, the virus manifests a wide range of symptoms. These can vary from mild symptoms (e.g., fever and cough) to severe conditions (respiratory failure, septic shock, or even death) [6]. 

The officially reported death toll reached 7 million by 2026, although estimates suggest that the actual number of deaths worldwide may have exceeded 10 million [7]. The latest SARS-CoV-2 variants currently in circulation as of April 2026, according to WHO variant tracking data, include JN.1 lineages, KP.2, and emerging recombinant variants [8]. COVID-19 has been shown to damage multiple tissues, including the olfactory epithelium, and its complex pathology makes it difficult to develop a comprehensive treatment, raising concerns regarding its eradication from the body [9]. Long COVID, also referred to as Post-Acute Sequelae of COVID-19 (PASC), describes the persistence or emergence of COVID-related symptoms following the acute phase of infection, even after apparent clinical recovery [10]. One study found that approximately 57% of patients exhibited at least one lingering symptom even one year after treatment for COVID-19 [11]. The most reported symptoms include dyspnea on exertion (34%), difficulty concentrating (32%), fatigue (31%), frailty (31%), and arthromyalgia (28%) [11]. Following recovery from acute COVID-19, individuals may develop multiple health complications affecting various organ systems, including pulmonary, cardiovascular, neurological, renal, endocrine, gastrointestinal, and integumentary systems [12]. Although COVID-19 has not been documented in space, the specific prevalence and incidence rates in microgravity remain theoretical. If COVID-19 were to occur in a spacecraft, the infection dynamics would differ from terrestrial settings due to unique microgravity and environmental factors. Observational data from spaceflight environments are currently limited. However, diseases such as Epstein–Barr Virus (EBV) infection have demonstrated increased virulence and prolonged survival under microgravity conditions [13].

Geographical factors may have played a role in shaping the spread and severity of COVID-19. For example, observed differences in hospitalization timing (e.g., 5.7 days in mainland China compared to 3.3 days in other regions) may reflect multiple confounding factors, including healthcare system infrastructure, testing availability, case ascertainment bias, and reporting practices, rather than intrinsic viral or population characteristics [14]. Additionally, reports of emerging COVID-19 strains between June and September 2020 in parts of the southern hemisphere, including Argentina, Australia, Brazil, and New Zealand [15], raise the possibility that geographic variation contributed to the evolution and behavior of the virus. Cold and dry environments are associated with higher transmission rates, whereas warm and humid conditions tend to slow the spread of pathogens [16]. Models incorporating several weather-related variables have been shown to describe epidemic trends more accurately than static models [17]. Cold and dry conditions during winter can suppress immune function, thereby amplifying the effects of COVID-19 [18]. 

Similar environmental conditions occur in high-altitude regions and aboard space stations. Additionally, reduced exposure to sunlight and lower vitamin D levels have been hypothesized to increase COVID-19 susceptibility; however, this relationship is complex and mediated by multiple immune and metabolic factors [19]. While some enclosed terrestrial environments show vitamin D deficiency, spacecrafts present distinct environmental characteristics. These include continuous artificial light cycles that differ from Earth’s day–night pattern, varying material, and microgravity-induced physiological changes that make direct terrestrial-to-space analogies speculative. Vitamin D deficiency may represent one of several potential environmental stressors influencing immune function during spaceflight; this requires empirical validation.

### 1.2. Space and Space Travel

Recognizing that multiple factors, including testing access, healthcare availability, socioeconomic status, and public health capacity, substantially influence disease burden, geographic variations in severity may reflect underlying differences in population immunity. However, this interpretation remains speculative and requires rigorous epidemiological validation. Fundamental questions regarding disease progression in extreme environments remain. Space is one of the most extreme environments. Space is defined as an area 100 km above the Earth’s surface [20]. Space travel has seen significant growth since its inception and is projected to expand in the future. As of 2021, 570 individuals have traveled to space and returned [21]. The peak year for human space travelers was 1985, when 63 individuals traveled to space. However, the COVID-19 pandemic significantly reduced space travel, with only 9, 12, and 7 individuals traveling to space in 2019, 2020, and 2021, respectively [1]. Although space travel has decreased in recent years, this trend is shifting. In 2026, NASA launched Artemis II, one of the most ambitious near-term human spaceflight programs, which is a 4-person mission spanning over 10 days. This mission represents a critical opportunity to assess infectious disease risk management for lunar exploration, with subsequent Artemis missions planning lunar landing operations in 2028 and beyond [22]. This highlights the growing demand for space travel in the post-pandemic era.

Space travel presents a unique challenge to human health. From a psychological standpoint, isolation and confinement during space missions can have significant psychological impacts. The small, enclosed environment of a spacecraft can become claustrophobic, potentially leading to interpersonal conflict [23]. Moreover, without the Earth’s atmospheric protection, astronauts are exposed to elevated levels of radiation. This radiation originates from the Earth’s magnetic field, solar particles, and galactic cosmic rays, increasing the risk of cancer and degenerative diseases, such as heart disease and cataracts [24]. In the absence of gravity, astronauts experience muscle atrophy and muscle mass deteriorates over time. Bone health is similarly affected, as astronauts lose between 1% and 1.5% of their bone mineral density per month in space [25]. This bone loss further increases the complications associated with long-term flights. When calcium is drained from the bones into the rest of the body, the kidneys bear the extra burden of excreting calcium, increasing the risk of developing kidney stones [26].

The effects of space travel extend to the immune system. Immune cells generated in space show altered gene expression. B cells, CD4+ T cells, CD8+ T cells, and CD14+ monocytes are all influenced by Differentially Expressed Genes (DEGs) [27]. Additionally, T cells (both CD4+ and CD8+) exhibit heightened responsiveness to infection, whereas B cells, which are essential for antibody production, are less responsive [28]. A more detailed study of immune function in space is currently limited because the effects of these DEGs return to normal shortly after astronauts return to Earth [27]. The microgravity environment in space can improve lung function. The cardiac output to the lungs increases by approximately 28%, enhancing gas absorption [29]. However, respiratory diseases remain a challenge during space missions. One notable respiratory disease outbreak occurred during the Apollo 7 mission when three astronauts contracted a respiratory infection that spread rapidly and disrupted mission cooperation [30]. A key complication is the accumulation of phlegm, which must be suctioned out owing to the absence of gravity [31]. Preventive measures, such as increasing the absolute humidity (AH) inside the spacecraft, may help reduce viral transmission. This approach is supported by findings from Earth, where higher AH levels are linked to decreased viral spread [32].

### 1.3. Research Questions

This study explored two key questions: First, what are the possible increased risks that COVID-19 poses to human health during space travel? Second, what preparedness measures can be implemented to prevent COVID-19 outbreaks? The absence of empirical data on COVID-19 transmission and virulence in microgravity creates an urgent need for evidence-based frameworks for space health. While direct experimentation in space environments is costly and logistically challenging, systematic comparative analysis of pathogens with documented health effects provides a pragmatic approach to identify potential risks and inform preparedness protocols before human exposure occurs. This methodology addresses a critical gap in current space health research.

To understand the potential risks of COVID-19 symptoms in space, it is important to explore their similarities with other airborne infectious diseases. Investigating the behavior of airborne infectious diseases in space environments, including those that have previously affected astronauts, is essential. By comparing the impact and viability of various diseases in space, the potential threat posed by COVID-19 can be estimated.

Developing a preparedness plan is crucial for preventing the risk of COVID-19 transmission in space. This involves reviewing existing guidelines for managing airborne diseases in space and determining whether these protocols align with those used on Earth. If so, similar measures could be adopted to address the transmission of COVID-19 during space missions. Drawing on previously established guidelines provides a structured framework for preventing the spread of the virus under the unique conditions of space.

Through systematic literature reviews, in accordance with the Preferred Reporting Standards for Systematic Reviews and Meta-Analyses (PRISMA) guidelines, we identified patterns and similarities between airborne infectious disease studies in space and COVID-19 to identify potential risks to astronauts. Comparisons were made between the effects of previously identified airborne diseases in space and on Earth to hypothesize how COVID-19 might behave in a microgravity environment. Our findings will help develop a comprehensive preparedness plan for COVID-19 prevention by integrating existing guidelines for the prevention of airborne infections in space and on Earth, along with Earth-based COVID-19 protocols, to create evidence-based measures for managing COVID-19 during space missions. 

## 2. Materials and Methods

### 2.1. Systematic Review of Studies of Airborne Infectious Diseases in Space

A systematic literature review was performed to identify studies examining airborne infectious diseases and their effects in spaceflight or microgravity environments. The search was conducted in April 2026 across PubMed and NASA’s Open Data Portal without temporal or language restrictions. To ensure comprehensive coverage, a structured search strategy was employed using (“airborne diseases” OR “airborne infection” OR “infectious” OR “pathogen transmission”) AND (“spaceflight” OR “microgravity” OR “space environment” OR “extraterrestrial”). Results were screened for relevance, and key journals were hand-searched to identify additional studies following PRISMA guidelines. 

#### 2.1.1. Inclusion and Exclusion Criteria

Studies were included if they were conducted in space, featured an airborne infectious disease or pathogen, or described health effects of any disease in a space environment. Due to the limited availability of relevant studies, the generalized effects of the identified diseases and pathogens were also considered. Secondary factors, such as primary research status or presentation of future safety guidelines, were noted during inclusion. However, these were not required for inclusion. Articles were excluded if they were inaccessible or written in a language that posed comprehension barriers and could not be translated, although no language-based filters were applied in the initial search. The selection process was facilitated by a standardized assessment method and a standardized assessment form (Figure S1).

#### 2.1.2. Data Extraction

Following the initial assessment, relevant and accessible articles were selected for data extraction. Extracted data included information on diseases, reported health effects, number of recorded cases (if available), and guidelines for managing these diseases in space. Data were initially collected using an extraction form, then organized into an Excel spreadsheet, with studies categorized by disease type (Figure S2).

#### 2.1.3. Collection of the Effects of Airborne Infectious Diseases on Earth

**Figure 1:**
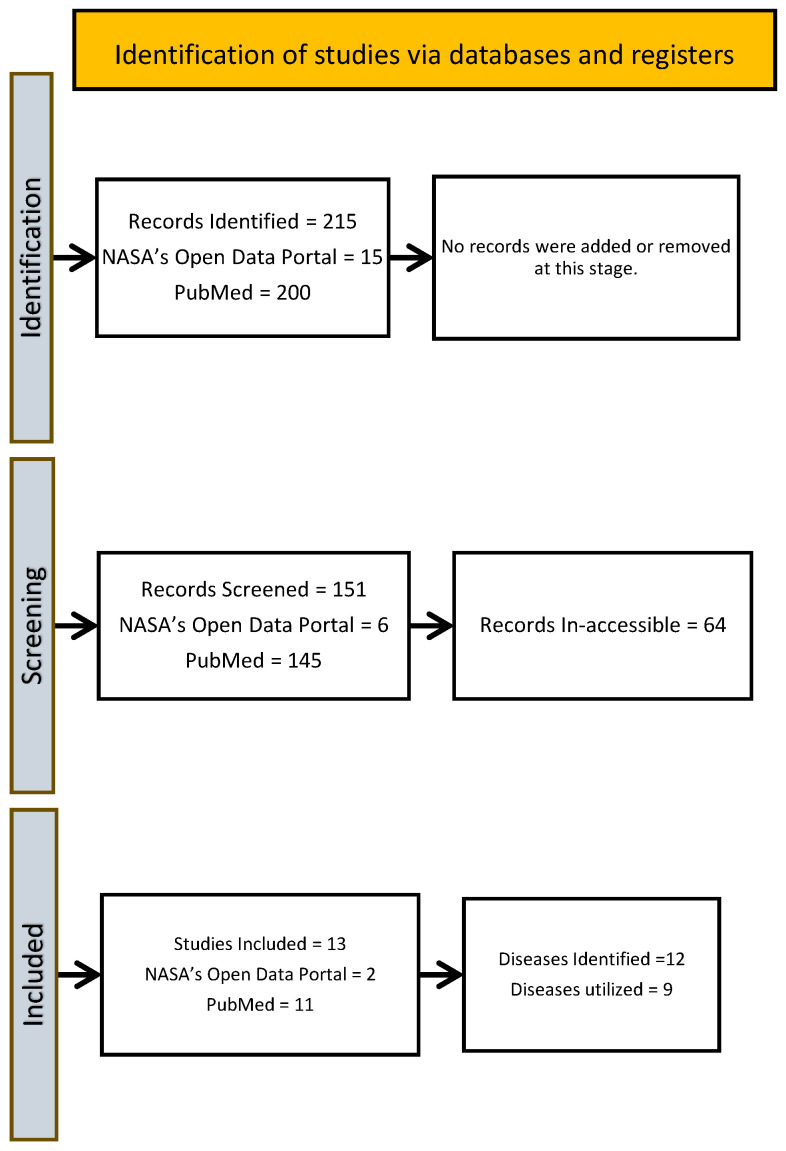
PRISMA flow diagram corresponding to Section 2.1 (Systematic Review), showing the study selection process for the literature on airborne infectious diseases in space, including identification, screening, eligibility, and inclusion of studies.

To ensure a fair comparison, space-based health effects were compared with known terrestrial effects. For standardization, reference data were obtained from Canadian Pathogen Safety Data Sheets: Infectious Substances. Three of the 12 diseases were excluded due to the absence of standardized Pathogen Safety Data Sheets (Figure S3). The PRISMA flow diagram is created to visualize the process (Figure 1).

### 2.2. Systematic Review for Studies That Link Airborne Infectious Diseases to COVID-19 on Earth

**Figure 2:**
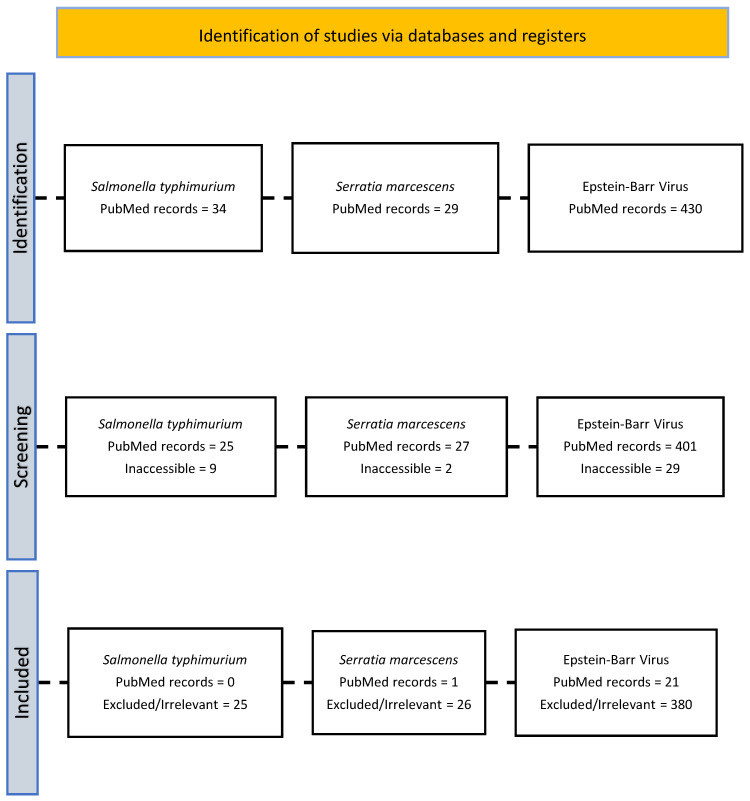
PRISMA flow diagram corresponding to Section 2.2 (Systematic Review), showing the selection process for studies examining associations between airborne infectious diseases (including Salmonella typhimurium, Serratia marcescens, and Epstein–Barr Virus) and COVID-19 on Earth.

A systematic literature review was conducted to identify articles reporting similarities between COVID-19 and other airborne infectious diseases identified in the initial review. The search identified nine systematic reviews for data extraction. PubMed was used for this systematic review. A time restriction was applied, and articles published before 2020 were excluded. The search strategy used (“COVID-19” OR “Coronavirus” OR “COVID”) AND [disease], where [disease] was systematically replaced with *Salmonella typhimurium*, *Serratia marcescens*, Varicella-Zoster Virus, Aspergillus fumigatus, Epstein–Barr Virus, Pseudomonas aeruginosa, *Escherichia coli*, *Staphylococcus aureus*, and *Klebsiella pneumoniae* (Figure S4). The search results were exported to an Excel spreadsheet, and accessible articles were stored as PDF files for reference. The PRISMA flow diagram is created to visualize the process (Figure 2).

#### 2.2.1. Inclusion and Exclusion Criteria

Studies were included if they discussed COVID-19 and a secondary disease with symptom overlap, regardless of pathophysiological similarities. Secondary factors, such as primary research status, were noted but were required for inclusion. Articles were excluded if inaccessible or untranslatable, although no language filters were in the initial search phase. Article selection was facilitated by a standardized assessment method and a standardized assessment form (Figure S5).

#### 2.2.2. Data Extraction

Following the assessment, relevant and accessible articles were screened for data extraction. Extracted data documented similarity between COVID-19 and secondary diseases. Data were collected using an extraction form organized into an Excel spreadsheet and categorized by diseases (Figure S6).

### 2.3. Estimating the Risk for COVID-19 in Space

To predict potential COVID-19 effects in space, a comparative approach was employed using data from airborne infectious diseases studied in terrestrial and microgravity environments.

Data from all three study components, space disease characteristics, terrestrial disease characteristics, and COVID-19 comparative effects, were analyzed using an Excel spreadsheet (Table S1). The analysis examined whether COVID-19 shares similar physiological impacts with known terrestrial diseases whose space-based effects have been documented, enabling extrapolation of COVID-19 pathophysiology, transmission, and risk profiles in microgravity. This comparative approach is based on the principle that pathogens with similar pathophysiological mechanisms to COVID-19 may exhibit analogous behavior under comparable extreme conditions. This approach has precedent in extreme environment risk assessment (e.g., deep-sea, high-altitude, polar research) and offers a cost-effective alternative to direct empirical space testing

Due to the absence of empirical SARS-CoV-2 data in microgravity, a comparative pathogenic analysis was performed. This approach is based on the principle that pathogens with similar pathophysiological mechanisms (e.g., respiratory droplet transmission and immune evasion) may exhibit analogous behavioral shifts under extreme spaceflight conditions.

### 2.4. Develop Preparedness Guidelines Against COVID-19 in Space

Current guidelines for managing airborne infectious diseases in space were reviewed. This review used the same 13 studies identified earlier to examine established protocols. Protocols were compiled and organized into a tabulated format as a structured reference for existing microgravity disease control measures (Table S2).

Terrestrial guidelines for managing airborne infectious diseases were systematically gathered into a database. Reference data were obtained from Canada’s Pathogen Safety Data Sheets: Infectious Substances and the *Canadian Biosafety Handbook*, which provide standardized terrestrial disease management protocols. Subsequently, existing COVID-19 Earth-based guidelines were incorporated into the database to enable direct comparison with other airborne disease protocols. Canada’s Pathogen Safety Data Sheets: Infectious Substances for COVID-19 served as key references. 

The comparative analysis examined three datasets: space-based disease protocols, terrestrial airborne disease guidelines, and COVID-19-specific guidelines. To ensure consistency in guideline identification, all pathogens were classified by risk group and containment level using Canada’s Pathogen Safety Data Sheets. Guidelines corresponding to each classification were sourced from the *Canadian Biosafety Handbook*. These classifications standardized the containment and response measures evaluated. COVID-19 was classified as Risk Group 2, Containment Level 2 per Canada’s Pathogen Safety Data Sheets. Analysis of these three sources identified potential COVID-19 prevention guidelines for space missions. 

### 2.5. Quality Assessment

This systematic review was conducted following PRISMA (Preferred Reporting Items for Systematic Reviews and Meta-Analyses) guidelines. Study quality and bias risks were assessed using the Critical Appraisal Skills Program (CASP) Checklist. The CASP Checklist was applied to evaluate methodological rigor. Studies were included if they met a minimum threshold of 8 out of 10 CASP criteria, ensuring methodological rigor.

## 3. Results

### 3.1. Systematic Review for Studies of Airborne Infectious Diseases in Space

The systematic review yielded 215 studies, which were screened by title and abstract according to inclusion criteria. Database searches were conducted using PubMed and NASA’s Open Data Portal. Of the 215 studies, 30% (64) were excluded due to inaccessibility (lack of digital availability, language barriers, or paywall restrictions). The remaining 151 studies were assessed for eligibility, of which 13 met the inclusion criteria for further analysis. 

From these 13 studies, 12 airborne infectious diseases were identified and their space-based effects were documented. However, only nine diseases had corresponding Canadian Pathogen Safety Data Sheets available for terrestrial effect comparison. Three diseases were excluded due to a lack of standardized terrestrial comparison data.

### 3.2. Systematic Review for Studies That Link Airborne Infectious Diseases to COVID-19 on Earth

**Figure 3:**
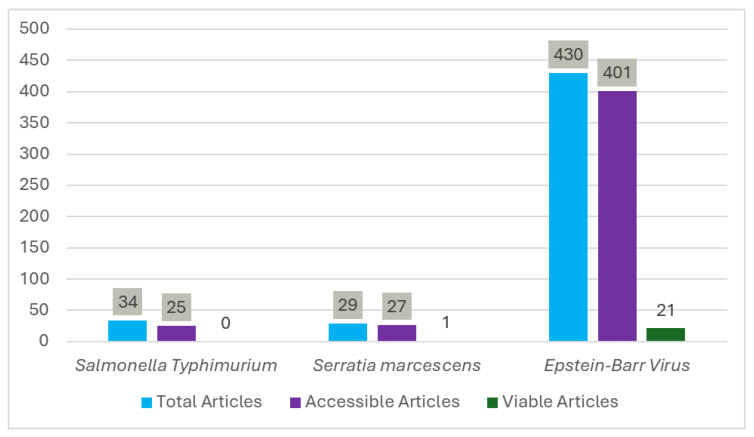
Availability of the literature on studies comparing COVID-19 to different pathogens. The number of studies from the systematic review used to establish a link between COVID-19 and airborne infectious diseases identified earlier. This figure compares the total, accessible, and viable research articles on Salmonella typhimurium, Serratia marcescens, and Epstein–Barr Virus. “Viable articles” were those that met the inclusion criteria.

Salmonella typhimurium, Serratia marcescens, and Epstein–Barr Virus (EBV) were selected for comparative analysis (Figure 3). Salmonella typhimurium, Serratia marcescens, and Epstein–Barr Virus (EBV) were selected for comparative analysis (Figure 3). For *Salmonella typhimurium*, 34 studies were identified; 25 were accessible. However, none met the inclusion criteria (lacking COVID-19, Salmonella typhimurium, or comparative data between them). For Serratia marcescens, 29 studies were identified; 27 were accessible. One study met the inclusion criteria, documenting a correlation between Serratia marcescens infection and COVID-19. For EBV, 430 studies were identified; 401 were accessible. Of these, 21 met criteria, demonstrating a correlation between EBV and COVID-19.

### 3.3. Estimate the Risk for COVID-19 in Space

Comparative analysis of COVID-19 with EBV and Serratia marcescens identified six potential COVID-19 effects in space: immune suppression and reactivation, inflammatory response, neurocognitive and systemic symptoms, gastrointestinal and pulmonary risk, increased virulence and growth potential, and greater severity and mortality risk (Table 1). These hypothesized effects are supported by findings that immune cells generated in space exhibit altered gene expression. B cells, CD4+ T cells, CD8+ T cells, and CD14+ monocytes show differential gene expression (DEG) in space [27].

### 3.4. Develop Preparedness Guidelines Against COVID-19 in Space

Guidelines for managing airborne infectious diseases in space were extracted from the 13 viable studies, which documented procedures and safety measures for infectious agents in space. These studies provide insights into the recommended procedures and safety measures implemented in space environments when dealing with infectious agents. Each pathogen was cross-referenced with Canada’s corresponding Pathogen Safety Data Sheet [33]. All pathogens were classified as Risk Group 2, requiring Containment Level 2 precautions. Containment guidelines for Risk Group 2 and Containment Level 2 were obtained from the *Canadian Biosafety Handbook*. Since all pathogens fell within this classification, space-based operational guidelines were presumed to align with Containment Level 2 standards. COVID-19 is classified as Risk Group 2 and Containment Level 2. Therefore, COVID-19 management guidelines for space should align with existing Risk Group 2 protocols.

For operational implementation, procedures were organized into four categories: preflight measures, in-flight prevention strategies, astronaut health and immune system support, and in-flight isolation and emergency response protocols (Table 2).

### 3.5. Clinical Implications of Identified Risks

The six theorized effects of COVID-19 in space (Table 1) represent distinct clinical scenarios requiring different mitigation strategies. Notably, three effects (immune suppression and reactivation, inflammatory response, and increased virulence) align with documented mechanisms in space-flown pathogens, strengthening biological plausibility. The remaining effects (neurocognitive and systemic symptoms, gastrointestinal and pulmonary risk, greater severity and mortality risk) represent speculative predictions requiring validation through targeted research. This differentiation enables mission planners to prioritize interventions based on evidence strength.

## 4. Discussion

This study was conducted over four months and utilized both publicly accessible databases and student access provided by the Kwantlen Polytechnic University Library to retrieve articles behind paywalls [34]. Nine airborne infectious diseases studied in microgravity environments were identified. Of these nine, Salmonella typhimurium, Serratia marcescens, and Epstein–Barr Virus (EBV) were selected for comparative analysis. Salmonella typhimurium and Serratia marcescens were selected due to the limited available literature, while EBV was chosen for its viral characteristics. Additionally, COVID-19 patients exhibit an increased incidence of EBV reactivation, further strengthening the association between these two viruses [35]. Six potential COVID-19 effects in space were identified from the comparative analysis. Two effects-immune suppression and reactivation and neurocognitive and systemic symptoms-were derived from the EBV-COVID-19 comparison. EBV reactivation in space suppresses immune function and affects multiple organ systems [13]. The prevalence of PASC conditions (including long COVID) with persistent or recurrent symptoms beyond four weeks further supports this association [36]. Increased virulence and growth potential was identified from the COVID-19–Serratia marcescens relationship, supported by demonstrated increased Serratia marcescens virulence under spaceflight conditions [37]. Three effects derived from both EBV and Serratia marcescens patterns: inflammatory response, gastrointestinal and pulmonary risks, and increased severity and mortality risk [38] Given EBV’s similar inflammatory responses and gastrointestinal and pulmonary risks in space, COVID-19 may exhibit analogous behavior in microgravity environments [13]. 

Although guidelines were initially collected generically for airborne infectious diseases in space, all identified pathogens were classified as Containment Level 2 and Risk Group 2. COVID-19 shares these same Containment Level 2 and Risk Group 2 classifications [39]. COVID-19 prevention guidelines were modeled by combining Containment Level 2 and Risk Group 2 recommendations from the *Canadian Biosafety Handbook* with existing space-based airborne disease protocol. Comparison with NASA’s Space Flight Human System Standard revealed that most proposed guidelines were already in place. This validates the methodology and demonstrates alignment with current best practices.

This systematic review demonstrates a comparative pathogenic analysis approach applicable to infectious disease threats in space and other extreme environments (e.g., Antarctic research stations, submarine crews, deep-sea operations). By identifying biological mechanisms predicting disease behavior in microgravity, this framework enables proactive risk assessment without direct human pathogen exposure in space. This becomes critical as commercial space tourism expands and introduces immunologically naive populations to spaceflight. Furthermore, enhanced air filtration, immune monitoring, and isolation protocols may drive aerospace innovations applicable to terrestrial healthcare (e.g., ICU design and epidemiological containment). This study bridges multiple disciplines with implications beyond space medicine.

This study had several limitations. The limited timeframe restricted database selection. The use of databases was restricted because of the short timeframe available to conduct the study. Given the exploratory scope, only a limited number of databases were consulted. Study selection was limited by the scarcity of the literature on airborne infectious diseases in space. Due to resource constraints, only a few researchers reviewed articles, potentially affecting review independence. Despite these constraints, this study provides a foundation for future research employing larger multi-reviewer teams to ensure objectivity. 

Although time constraints limited database selection and disease comparisons, the risk assessment framework remains valid. These limitations identify priority areas for future research. Specifically, including varicella-zoster virus and Mycobacterium tuberculosis with documented spaceflight reactivation potential [40] would strengthen predictions of viral and bacterial threats. Prospective studies examining immune biomarkers in post-mission astronauts would empirically validate theorized immune suppression mechanisms. Future research should investigate whether the six effects would manifest differently in near-Earth orbit (ISS) versus deep-space environments due to radiation exposure and mission duration variations.

This approach enables risk estimation without direct sampling or outbreak occurrence. The methodology uses disease characteristics from extreme environments, compares terrestrial effects, and applies a comparative analysis to diseases of interest. This enables accurate risk analysis and understanding of extreme environment health effects. This methodology is applicable to health risk analysis in other extreme or inaccessible environments. By addressing these gaps, this research contributes to understanding infectious disease behavior in unique environments and supports safer, more sustainable space habitation. Recent studies demonstrate increased microorganism virulence in microgravity environments, underscoring the need for continued research [41].

This study establishes a foundation for evidence-based space mission health planning using systematic comparative analysis. As human space exploration accelerates, these frameworks become increasingly essential. Space agencies should formally integrate infectious disease risk assessments into mission planning and prioritize empirical validation studies for theorized effects. International collaboration and data sharing among space agencies regarding crew health incidents are critical for developing comprehensive preparedness guidelines. The methodological framework should be applied to emerging pathogens (e.g., monkeypox and novel influenza strains) to build a predictive database for space pathogen behavior.

## Figures and Tables

**Table 1:** Theorized clinical hypotheses for the effects of COVID-19 in space based on comparisons with EBV and Serratia marcescens. This table outlines six potential health impacts of COVID-19 in space, derived from observed similarities with the behavior of EBV and Serratia marcescens under spaceflight or microgravity conditions. These effects include immune suppression and reactivation, heightened inflammatory responses, neurocognitive and systemic symptoms, gastrointestinal and pulmonary risks, increased virulence and growth potential, and greater severity and mortality risks.

Immune Suppression and Reactivation:	Similar to EBV, COVID-19 may exhibit increased reactivation or persistence in space due to spaceflight-induced immune suppression, leading to prolonged viral activity and an increased risk of complications, such as inflammation or post-viral syndromes.
Inflammatory Response:	As seen with both EBV and Serratia marcescens, COVID-19 may trigger heightened inflammatory responses in space, potentially worsening outcomes such as fever, fatigue, and organ involvement (e.g., lungs and liver).
Neurocognitive and Systemic Symptoms:	Based on the similarities between EBV’s associations with fatigue, brain fog, myalgia, and cognitive dysfunction, COVID-19 in space may similarly lead to LC-like symptoms affecting the neurological and muscular systems.
Gastrointestinal and Pulmonary Risk:	Similar to EBV’s role in gastrointestinal inflammation and Serratia’s pulmonary risks, COVID-19 may also result in exacerbated GI and respiratory issues in space.
Increased Virulence and Growth Potential:	As Serratia marcescens demonstrates enhanced virulence due to altered bacterial growth kinetics in microgravity, COVID-19’s replication rate and tissue tropism could be altered, potentially amplifying disease severity.
Greater Severity and Mortality Risk:	Given that both EBV and Serratia infections are associated with worse clinical outcomes in space or co-infection settings, COVID-19 in space may lead to greater severity, especially in critically ill or immunocompromised astronauts.

**Table 2:** Recommended COVID-19 prevention and response strategies for space missions. The table outlines comprehensive measures, including preflight vaccination and screening, in-flight environmental and health monitoring, immune system support, and isolation protocols. These are complementary to the current NASA guidelines.

Preflight Measures:	Vaccination: Ensure that astronauts are vaccinated against COVID-19 before traveling to space. Screening: Astronauts should be tested for COVID-19 before embarking on a mission. This includes regular checks for symptoms and testing, as needed. Health Monitoring: Continuous health checks, including temperature and symptom screening, should be performed before and during the mission.
In-Flight Prevention:	Air and Water Systems: Ensure that spacecraft have proper filtration systems to prevent airborne pathogens and maintain clean water systems. Medical Support: Telemedicine should be available for consultation with Earth-based doctors if an astronaut shows symptoms of COVID-19. Decontamination: Routine cleaning and disinfection of surfaces in spacecraft and equipment should be conducted using appropriate procedures and PPE.
Astronaut Health and Immune Support:	Immune Function Monitoring: Astronauts’ immune health should be regularly monitored to detect any changes that might increase the risk of infection. Nutritional Support: Astronauts should be provided with dietary supplements, including probiotics, to support immune function and prevent infections. Mental Health: Ensure that astronauts have mental health support to manage stress, which can affect the immune system.
In-Flight Isolation and Response:	Isolation Areas: If an astronaut shows signs of infection, they should be isolated in a designated area within the spacecraft. Environmental Controls: Regular microbial monitoring of the spacecraft environment is essential, especially for airborne viruses such as SARS-CoV-2. Emergency Response: Prepare for contingencies, such as extended isolation or quarantine, if an outbreak occurs.

## Data Availability

Data stored in our Institution. It is available upon request.

## Supplementary Materials

The following supporting information is available at https://www.mdpi.com/article/10.3390/pathogens15050498/s1. Figure S1: Assessment form used for screening articles based on eligibility criteria. The form includes organizational aspects such as article ID, author, journal/source, title, and publication type. It also assesses study characteristics, including location, disease type, health effects, study type, and future guidelines. Eligibility criteria are evaluated with “Yes,” “No,” or “Maybe” checkboxes, along with a section for noting the location of relevant information in the text; Figure S2: Data extraction form used for systematically collecting relevant information from selected articles. The form includes fields for article ID, title, author, study design, disease type, year of disease occurrence, health effects, number of reported cases, and guidelines implemented for disease management; Figure S3: Flowchart depicting the systematic review process for identifying, screening, and selecting airborne infectious disease studies in space. The diagram illustrates the number of records retrieved from NASA’s Open Data Portal and PubMed, the numbers of accessible records, viable articles, and identified diseases included in the study. The exclusion of three diseases was due to the absence of standardized Pathogen Safety Data Sheets; Figure S4: Number of articles retrieved from PubMed for each airborne infectious disease in comparison to COVID-19. The search strategy included the combination of COVID-19 with each listed disease, resulting in varying numbers of relevant articles. Escherichia coli and Staphylococcus aureus had the highest number of retrieved studies, while Serratia marcescens had the lowest; Figure S5: Assessment form used for screening articles comparing COVID-19 with other infectious diseases. The form includes organizational details such as article ID, author, journal/source, and publication type. It evaluates study characteristics based on eligibility criteria, including the presence of COVID-19, a secondary disease, health effects for both diseases, and whether the study is a primary research article. Eligibility is determined using “Yes,” “No,” or “Maybe” checkboxes, with a section for noting the location of relevant information in the text. Figure S6: Data extraction form used for systematically collecting information on the relationship between COVID-19 and other infectious diseases. The form includes fields for article ID, title, author, study design, and a dedicated section to document the identified similarities and relationships between COVID-19 and the secondary disease under investigation; Table S1: Comparison of health effects of airborne infectious diseases in space and on Earth, and their relevance to COVID-19. This table summarizes the known effects of 12 airborne pathogens in both space and Earth environments, including Salmonella typhimurium, Serratia marcescens, Varicella-Zoster Virus, Aspergillus fumigatus, Epstein–Barr Virus, Pseudomonas aeruginosa, Escherichia coli, Staphylococcus aureus, and Klebsiella pneumoniae. It highlights similarities in virulence, immune response, and symptom overlap with COVID-19, where applicable. The table also notes where reviews were not conducted due to time constraints or limited data availability; Table S2: Comparison of guidelines for managing airborne infectious diseases in space and on Earth, including COVID-19. This table summarizes key recommendations from studies on airborne pathogens in space, standard Earth-based biosafety measures, and existing COVID-19 guidelines. It includes containment protocols, infection prevention strategies, immune system monitoring, microbial surveillance, and therapeutic considerations. Guidelines were also aligned using the *Canadian Biosafety Handbook* based on Containment Level 2 and Risk Group 2 classifications; Table S3: PRISMA 2020 Checklist of this study.

## References

[1] CSIS Aerospace Security Project (2022). Human Visits to Space per Year. Our World in Data. https://ourworldindata.org/grapher/annual-space-visits.

[2] Taveau J. NASA Accelerates Space Exploration, Earth Science for All in 2024. NASA. 6 December 2024. https://www.nasa.gov/news-release/nasa-accelerates-space-exploration-earth-science-for-all-in-2024/.

[3] Chakraborty I., Maity P. (2020). COVID-19 Outbreak: Migration, Effects on Society, Global Environment and Prevention. Sci. Total Environ..

[4] WHO Data WHO COVID-19 Dashboard. World Health Organization. 12 January 2020. https://data.who.int/dashboards/covid19/cases?n=c.

[5] World Health Organization Naming the Coronavirus Disease (COVID-19) and the Virus That Causes It. World Health Organization. https://www.who.int/emergencies/diseases/novel-coronavirus-2019/technical-guidance/naming-the-coronavirus-disease-%28covid-2019%29-and-the-virus-that-causes-it.

[6] Public Health Agency of Canada COVID-19: Symptoms, Treatment, What to Do If You Feel Sick. Canada.ca. 27 January 2023. https://www.canada.ca/en/public-health/services/diseases/2019-novel-coronavirus-infection/symptoms.html.

[7] World Health Organization The True Death Toll of COVID-19. World Health Organization. April 2022. https://www.who.int/data/stories/the-true-death-toll-of-covid-19-estimating-global-excess-mortality.

[8] World Health Organization Tracking SARS-CoV-2 Variants. World Health Organization. 2 December 2024. https://www.who.int/activities/tracking-SARS-CoV-2-variants/?form=MG0AV3.

[9] Edalatifard M., Rahimi B., Vesal A. (2020). Coronavirus and Its Effect on the Respiratory System: Is There Any Association between Pneumonia and Immune Cells. J. Fam. Med. Prim. Care.

[10] Diaz J.V., Soriano J.B. (2021). A Delphi Consensus to Advance on a Clinical Case Definition for Post COVID-19 Condition: A WHO Protocol. Lancet Infect. Dis..

[11] Mudgal S.K., Gaur R., Rulaniya S., Latha T., Agarwal R., Kumar S., Varshney S., Sharma S., Bhattacharya S., Kalyani V. (2023). Pooled Prevalence of Long COVID-19 Symptoms at 12 Months and Above Follow-Up Period: A Systematic Review and Meta-Analysis. Cureus.

[12] CDC Signs and Symptoms of Long COVID. Centers for Disease Control and Prevention. 17 September 2024. https://www.cdc.gov/covid/long-term-effects/long-covid-signs-symptoms.html?form=MG0AV3.

[13] Mehta S., Bloom D., Plante I., Stowe R., Feiveson A.H., Renner A., Dhummakupt A., Markan D., Zhang Y., Wu H. (2018). Reactivation of Latent Epstein-Barr Virus: A Comparison After Exposure to Gamma, Proton, Carbon, and Iron Radiation. Int. J. Mol. Sci..

[14] Li J., Huang D.Q., Zou B., Yang H., Hui W.Z., Rui F., Yee N.T.S., Liu C., Nerurkar S.N., Kai J.C.Y. (2020). Epidemiology of COVID-19: A Systematic Review and Meta-Analysis of Clinical Characteristics, Risk Factors, and Outcomes. J. Med. Virol..

[15] Araújo M.B., Naimi B. (2020). Spread of SARS-CoV-2 Coronavirus Likely Constrained by Climate. medRxiv.

[16] Mecenas P., Bastos R.T., Vallinoto A.C., Normando D. (2020). Effects of Temperature and Humidity on the Spread of COVID-19: A Systematic Review. PLoS ONE.

[17] Chen B., Liang H., Yuan X., Hu Y., Xu M., Zhao Y., Zhang B., Tian F., Zhu X. (2020). Roles of Meteorological Conditions in COVID-19 Transmission on a Worldwide Scale. medRxiv.

[18] Lowen A.C., Mubareka S., Steel J., Palese P. (2007). Influenza Virus Transmission Is Dependent on Relative Humidity and Temperature. PLoS Pathog..

[19] Cannell J.J., Vieth R., Umhau J.C., Holick M.F., Grant W.B., Madronich S., Garland C.F., Giovannucci E. (2006). Epidemic Influenza and Vitamin D. Epidemiol. Infect..

[20] Hurley S. Where Does Space Begin? Explaining Science. 8 January 2019. https://explainingscience.org/2019/01/01/where-does-space-begin/.

[21] CSIS Aerospace Security Project (2022). Cumulative Number of Human Visits to Space. Our World in Data. https://ourworldindata.org/grapher/cumulative-space-visits?country=OWID_WRL~JPN~RUS~CAN~USA.

[22] Massengill D. Artemis II. NASA. 10 April 2023. https://www.nasa.gov/mission/artemis-ii/.

[23] Liu Q., Zhou R., Zhao X., Chen X., Chen S. (2016). Acclimation During Space Flight: Effects on Human Emotion. Mil. Med. Res..

[24] Montesinos C.A., Khalid R., Cristea O., Greenberger J.S., Epperly M.W., Lemon J.A., Boreham D.R., Popov D., Gorthi G., Ramkumar N. (2021). Space Radiation Protection Countermeasures in Microgravity and Planetary Exploration. Life.

[25] Sibonga J.D., Evans H.J., Smith S.A., Spector E.R., Yardley G. (2017). Risk of Bone Fracture Due to Spaceflight-Induced Changes to Bone. NASA. https://humanresearchroadmap.nasa.gov/Evidence/reports/Fracture.pdf.

[26] Smith S.M., Heer M., Shackelford L.C., Sibonga J.D., Spatz J., Pietrzyk R.A., Hudson E.K., Zwart S.R. (2015). Bone Metabolism and Renal Stone Risk During International Space Station Missions. Bone.

[27] Jones C.W., Overbey E.G., Lacombe J., Ecker A.J., Meydan C., Ryon K., Tierney B., Damle N., MacKay M., Afshin E.E. (2024). Molecular and Physiological Changes in the SpaceX Inspiration4 Civilian Crew. Nature.

[28] Overbey E.G., Kim J., Tierney B.T., Park J., Houerbi N., Lucaci A.G., Medina S.G., Damle N., Najjar D., Grigorev K. (2024). The Space Omics and Medical Atlas (SOMA) and International Astronaut Biobank. Nature.

[29] Prisk G.K. (2019). Pulmonary Challenges of Prolonged Journeys to Space: Taking Your Lungs to the Moon. Med. J. Aust..

[30] Pavlečić B., Runzheimer K., Siems K., Koch S., Cortesão M., Ramos-Nascimento A., Moeller R. (2022). Spaceflight Virology: What Do We Know About Viral Threats in the Spaceflight Environment?. Astrobiology.

[31] Ostovar M. About Apollo 7, the First Crewed Apollo Space Mission. NASA. 10 October 2023. https://www.nasa.gov/missions/apollo/about-apollo-7-the-first-crewed-apollo-space-mission/.

[32] Wang J., Tang K., Feng K., Lin X., Lv W., Chen K., Wang F. (2021). Impact of Temperature and Relative Humidity on the Transmission of COVID-19: A Modelling Study in China and the United States. BMJ Open.

[33] Public Health Agency of Canada Pathogen Safety Data Sheets. Canada.ca. 5 February 2025. https://www.canada.ca/en/public-health/services/laboratory-biosafety-biosecurity/pathogen-safety-data-sheets-risk-assessment.html.

[34] Kwantlen Polytechnic University KPU Library. https://www.kpu.ca/library.

[35] Bernal K.D., Whitehurst C.B. (2023). Incidence of Epstein-Barr Virus Reactivation Is Elevated in COVID-19 Patients. Virus Res..

[36] Greenhalgh T., Sivan M., Perlowski A., Nikolich J.Z. (2024). Long COVID: A Clinical Update. Lancet.

[37] Gilbert R., Torres M., Clemens R., Hateley S., Hosamani R., Wade W., Bhattacharya S. (2020). Spaceflight and Simulated Microgravity Conditions Increase Virulence of Serratia marcescens in the Drosophila melanogaster Infection Model. npj Microgravity.

[38] Rousseau B.A., Bhaduri-McIntosh S. (2023). Inflammation and Epstein–Barr Virus at the Crossroads of Multiple Sclerosis and Post-Acute Sequelae of COVID-19 Infection. Viruses.

[39] Public Health Agency of Canada Severe Acute Respiratory Syndrome Coronavirus-2 (SARS-CoV-2): Pathogen Safety Data. Canada.ca. 13 April 2023. https://www.canada.ca/en/public-health/services/laboratory-biosafety-biosecurity/pathogen-safety-data-sheets-risk-assessment/severe-acute-respiratory-syndrome-coronavirus-2.html.

[40] Martínez-Reviejo R., Tejada S., Adebanjo G.A., Chello C., Machado M.C., Parisella F.R., Campins M., Tammaro A., Rello J. (2022). Varicella-Zoster Virus Reactivation Following Severe Acute Respiratory Syndrome Coronavirus 2 Vaccination or Infection: New Insights. Eur. J. Intern. Med..

[41] Huss P., Chitboonthavisuk C., Meger A., Nishikawa K., Oates R.P., Mills H., Holzhaus O., Raman S. (2026). Microgravity reshapes bacteriophage–host coevolution aboard the international space station. PLoS Biol..

