# Supplementary material for: COVID-19 in Space: Possible Health Risks and Preparedness Guidelines
Source: Pathogens. 2026 May 6;15(5):498. doi: 10.3390/pathogens15050498 (PMC13209358; doi:10.3390/pathogens15050498)
Supplement: Supplementary file 1 [file pathogens-15-00498-s001.zip › pathogens-4230190-supplementary-updated.pdf]

Assessment Form

**Organizational Aspects:**	Article ID:	☐ Included	☐ Included
**Author; Year**			
**Journal/Source**			
**Article Title**			
**Publication Type**			
**Notes**			

**Study Characteristics**		**Eligibility Criteria**	**Eligibility Criteria Met?**			**Location in Text**
			**Yes**	**No**	**Maybe**	
**1**	Location	In Space	☐	☐	☐	
**2**	Disease	Airborne Infectious	☐	☐	☐	
**3**	Health Effects	Present	☐	☐	☐	
**4**	Study Type	Primary Study	☐	☐	☐	
**5**	Future Guidelines	Present/Referenced	☐	☐	☐	

Figure S1: Assessment form used for screening articles based on eligibility criteria. The form includes organizational aspects such as article ID, author, journal/source, title, and publication type. It also assesses study characteristics, including location, disease type, health effects, study type, and future guidelines. Eligibility criteria are evaluated with "Yes," "No," or "Maybe" checkboxes, along with a section for noting the location of relevant information in the text.

Data Extraction Form:

Criteria	Information
**Article ID**	
**Title**	
**Author**	
**Study Design**	
**Disease**	
**Year of Disease**	
**Health Effects of Disease**	
**Number of Cases**	
**Guidelines put in place**	

Figure S2: Data extraction form used for systematically collecting relevant information from selected articles. The form includes fields for article ID, title, author, study design, disease type, year of disease occurrence, health effects, number of reported cases, and guidelines implemented for disease management.

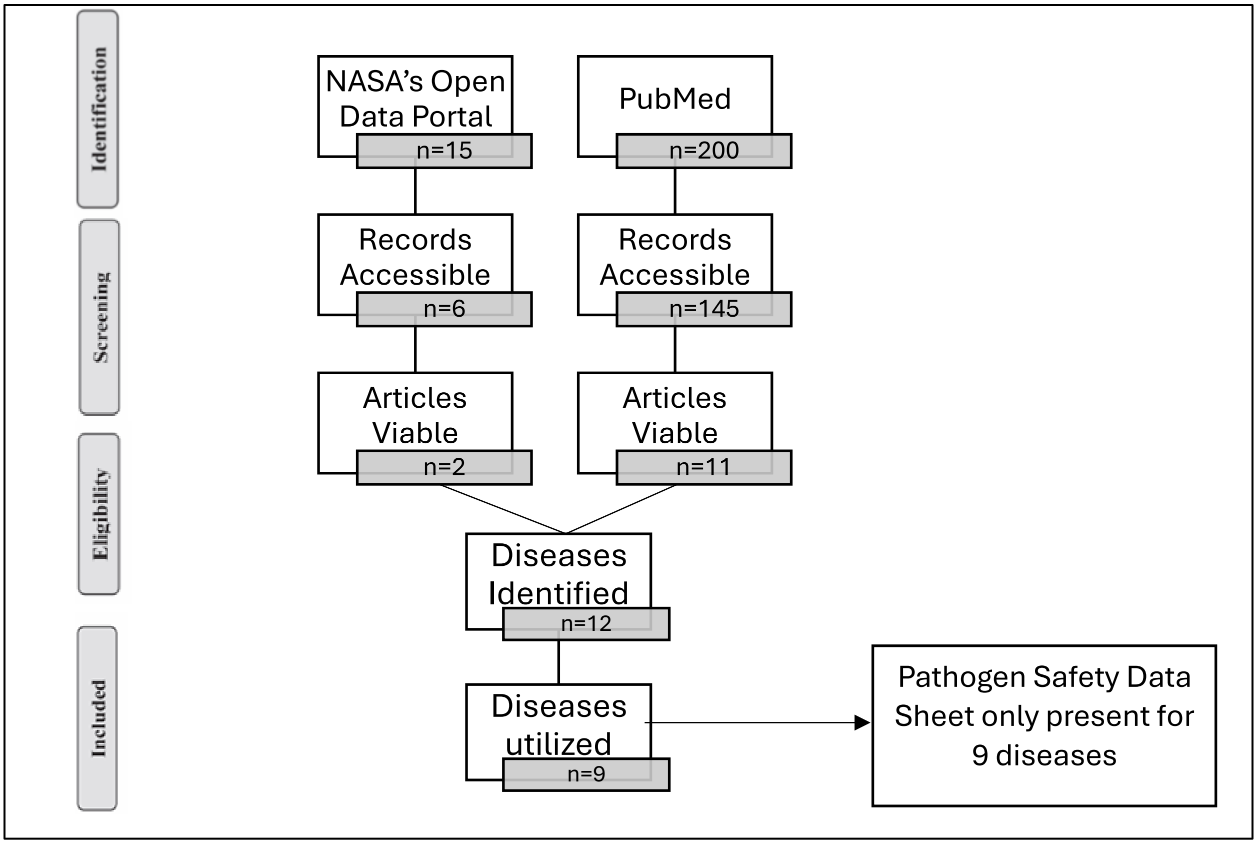

Figure S3: Flowchart depicting the systematic review process for identifying, screening, and selecting studies on airborne infectious diseases in space. The diagram illustrates the number of records retrieved from NASA's Open Data Portal and PubMed, the number of accessible records, viable articles, identified diseases, and the final number of diseases included in the study. The exclusion of three diseases was due to the absence of standardized Pathogen Safety Data Sheets.

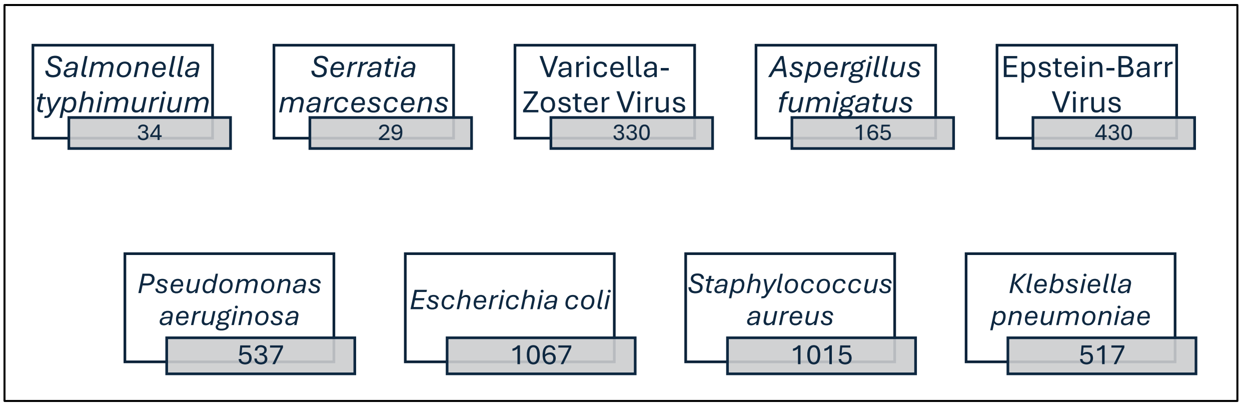

Figure S4: **Number of articles retrieved from PubMed for each airborne infectious disease in comparison to COVID-19**. The search strategy included the combination of COVID-19 with each listed disease, resulting in varying numbers of relevant articles. *Escherichia coli* and *Staphylococcus aureus* had the highest number of retrieved studies, while *Serratia marcescens* had the lowest.

Assessment Form

**Organizational Aspects:**	Article ID:	☐ Included	☐ Excluded
**Author; Year**			
**Journal/Source**			
**Article Title**			
**Publication Type**			
**Notes**			

**Study Characteristics**		**Eligibility Criteria**	**Eligibility Criteria Met?**			**Location in Text**
			**Yes**	**No**	**Maybe**	
**1**	Diseases 1	COVID-19	☐	☐	☐	
**2**	Disease 2		☐	☐	☐	
**3**	Health Effects	Present for both	☐	☐	☐	
**4**	Study Type	Primary Study	☐	☐	☐	

Figure S5: Assessment form used for screening articles comparing COVID-19 with other infectious diseases. The form includes organizational details such as article ID, author, journal/source, and publication type. It evaluates study characteristics based on eligibility criteria, including the presence of COVID-19, a secondary disease, health effects for both diseases, and whether the study is a primary research article. Eligibility is determined using "Yes," "No," or "Maybe" checkboxes, with a section for noting the location of relevant information in the text.

Data Extraction Form:

Criteria	Information
**Article ID**	
**Title**	
**Author**	
**Study Design**	
**Relationship between COVID and -disease-**	

Figure S6: Data extraction form used for systematically collecting information on the relationship between COVID-19 and other infectious diseases. The form includes fields for article ID, title, author, study design, and a dedicated section to document the identified similarities and relationships between COVID-19 and the secondary disease under investigation.

Table S1: Comparison of health effects of airborne infectious diseases in space and on Earth, and their relevance to COVID-19. This table summarizes the known effects of 12 airborne pathogens in both space and Earth environments, including *Salmonella typhimurium, Serratia marcescens, Varicella-Zoster Virus, Aspergillus fumigatus, Epstein-Barr Virus, Pseudomonas aeruginosa, Escherichia coli, Staphylococcus aureus, and Klebsiella pneumoniae*. It highlights similarities in virulence, immune response, and symptom overlap with COVID-19, where applicable. The table also notes where reviews were not conducted due to time constraints or limited data availability.

Sr. No.	Disease	Effects in Space	Effects on Earth	Comparison to COVID-19
1	Salmonella Typhimurium infection	Increased virulence and stress resistance of Salmonella in spaceflight conditions.<br><br>Altered immune and gastrointestinal responses in human intestinal epithelial cells<br><br>Potential for heightened infection risk during space missions.<br><br>Space-grown strains showed hypervirulence in murine models.<br><br>Increased biofilm formation under low-shear microgravity conditions.<br><br>S. Typhimurium exhibited increased virulence and stress resistance in spaceflight conditions<br><br>Enhanced pathogen colonization and altered transcriptional responses in infected human cells<br><br>Spaceflight altered host immune responses, including reduced TLR4 expression, which may impair immune recognition of Salmonella<br><br>Infection triggered higher pro-inflammatory responses in spaceflight-exposed human cells compared to ground controls	Salmonella Typhimurium commonly causes gastroenteritis, which begins suddenly with nausea, vomiting, abdominal cramps, diarrhea, headache, chills, and fever up to 39°C.<br><br>The symptoms of gastroenteritis usually last between 5 to 7 days and can range from mild to severe.<br><br>It is responsible for approximately 1.3 billion cases and 3 million deaths globally each year, including 1.4 million cases and 600 deaths in the U.S. annually.<br><br>In about 3–10% of infected people, the infection progresses to bacteremia, where the bacteria enter the bloodstream.<br><br>Bacteremia can lead to septic shock and endocarditis, particularly in people over 50 or those with existing heart conditions.<br><br>It can also cause infections of the aorta, liver, spleen, and biliary tract, especially in those with structural abnormalities.<br><br>Other complications include mesenteric lymphadenitis, osteomyelitis (especially in long bones and the spine), urinary tract infections, pneumonia, pulmonary or brain abscesses, subdural and epidural empyema, and meningitis (rarely).<br><br>In severe cases, death may occur as a result of bacteremia.<br><br>People with weakened immune systems or pre-existing conditions like HIV/AIDS, diabetes, cancer, cirrhosis, chronic granulomatous disease, or sickle cell disease are at higher risk of developing these serious complications.	No Connection Established
2	Serratia marcescens infection	Increased virulence of Serratia marcescens under spaceflight and microgravity conditions, leading to decreased survival rates.<br><br>The increased lethality is linked to bacterial growth kinetics rather than changes in host immune response.<br><br>Increased virulence of Serratia marcescens when grown in spaceflight conditions, leading to decreased survival rates in Drosophila melanogaster.	Serratia marcescens is an opportunistic pathogen and one of the top ten causes of bacteremia in North America.<br><br>It can cause a wide range of infections including bacteremia, pneumonia, intravenous catheter-associated infections, osteomyelitis, endocarditis, and in rare cases, endophthalmitis (an eye infection).<br><br>Endophthalmitis symptoms appear rapidly and may include fever, redness, ocular pain, swelling around the eyes, and pus in the eye (hypopyon).	Infections can lead to bacterial cross-contamination<br><br>Increased risk of S. marcescens superinfection<br><br>Ventilator-associated pneumonia and septic shock.

		The increased lethality was linked to changes in bacterial growth kinetics rather than host immune suppression.	The mortality rate due to Serratia bacteremia can reach 37% six months post-infection.<br><br>Other rare neonatal infections include otitis externa, enterocolitis, omphalitis, gastroenteritis, septic arthritis, and intraperitoneal abscesses.<br><br>Risk factors in neonates include low birth weight, use of mechanical ventilation, and premature birth (under 37 weeks).<br><br>The mortality rate in neonates with Serratia infection is reported to be as high as 44%.<br><br>Most infections are caused by non-pigmented strains, which are more virulent than pigmented ones.	
3	Varicella-Zoster Virus (VZV)	Reactivation risk increases in spaceflight.<br><br>Higher viral load in astronauts, increasing transmission potential.<br><br>Causes chickenpox (more easily spread) and shingles<br><br>Asymptomatic VZV reactivation was detected in astronauts.<br><br>Spaceflight stress suppressed cell-mediated immunity (CMI), leading to higher viral shedding.<br><br>VZV DNA was present in astronaut saliva during and after spaceflight, confirming reactivation without visible disease	Causes a generalized itchy, vesicular rash and fever, primarily in children.<br><br>Lesions typically begin on the back of the head and ears, spreading to the face, neck, trunk, and limbs.<br><br>The number of lesions can range from 10 to several hundred.<br><br>May lead to viremia and visceral organ involvement in immunocompromised individuals.<br><br>Can cause pneumonia, hepatitis, encephalitis, transient ataxia, aseptic meningitis, and transverse myelitis.<br><br>Thirty percent of children with leukemia may develop disseminated varicella, with a 7% mortality rate.<br><br>Less common complications include arthritis, glomerulonephritis, myocarditis, and purpura fulminans.<br><br>The virus becomes latent in sensory ganglia neurons after initial infection.<br><br>Reactivation of the virus leads to shingles (herpes zoster), especially in people over 50 years old.<br><br>Shingles causes a painful vesicular rash along the area of skin supplied by the affected sensory nerve.<br><br>Postherpetic neuralgia may occur, with pain lasting months or years after the rash heals.<br><br>In immunocompromised patients, reactivation can lead to disseminated infection involving visceral organs.	Review not conducted due to time constraints.
4	Aspergillus fumigatus	Causes aspergillosis, which is a serious infection in immunocompromised individuals or those with chronic lung conditions.<br><br>Space conditions may enhance its virulence	Aspergillus fumigatus is the most common cause of aspergillosis, a group of diseases caused by Aspergillus spp.<br><br>It can cause allergic reactions such as allergic bronchopulmonary aspergillosis, rhinitis, and farmer’s lung.	Review not conducted due to time constraints.

			It may result in superficial and local infections like cutaneous infections, otomycosis (ear infection), and tracheobronchitis.<br><br>Aspergilloma (fungal ball in the lungs) and osteomyelitis (bone infection) can occur in patients with damaged tissue.<br><br>Invasive pulmonary aspergillosis is the most severe form, primarily affecting immunocompromised individuals.<br><br>Invasive infections often start in the sinopulmonary tract, particularly the lungs.<br><br>Invasive sinusitis symptoms include fever, facial pain, headache, swelling, nosebleeds, proptosis, nerve issues, and palate ischemia.<br><br>Invasive pulmonary aspergillosis presents with fever, cough, dyspnea, and may include pleural chest pain due to vascular invasion.<br><br>If left untreated, the infection can spread through the bloodstream to other organs.<br><br>Central nervous system involvement can lead to seizures or stroke.<br><br>The mortality rate in immunocompromised patients can be significant, especially without early treatment.<br><br>High-risk groups include patients with acute leukemia, transplant recipients, and those with neutropenia or graft-versus-host disease.	
5	Beauveria bassiana	Can cause disseminated infections, though it is rarely a human pathogen	Pathogen Safety Data Sheet Not Available	Review not conducted due to time constraints.
6	Roseolovirus (Human Herpesvirus 6 & 7)	Known to cause fever, rash, and neurological complications.<br><br>Potential reactivation in astronauts due to spaceflight-induced immune suppression	Pathogen Safety Data Sheet Not Available	Review not conducted due to time constraints.
7	Epstein-Barr Virus (EBV)	Causes infectious mononucleosis (mono).<br><br>Increased viral shedding in saliva under spaceflight conditions<br><br>Space radiation exposure led to reactivation of latent EBV, increasing viral load in astronauts.<br><br>Lytic gene transcription was induced at all radiation doses, with the highest activation at day 4 post-exposure.<br><br>EBV reactivation is linked to immune suppression and may increase the risk of lymphoproliferative diseases and B-cell malignancies.	Most childhood EBV infections are asymptomatic.<br><br>EBV can cause infectious mononucleosis.<br><br>Symptoms of infectious mononucleosis include fever, sore throat, pharyngitis, tonsillitis, lymphadenopathy, and fatigue.<br><br>Rashes may occur, especially after taking ampicillin or amoxicillin.<br><br>Fatigue can persist for up to a year in some patients.	Participants with long COVID showed elevated antibody responses to EBV antigens, especially p23 and gp42, suggesting EBV reactivation. This reactivation is linked to symptoms such as fatigue and immune dysfunction, which are also seen in long COVID, indicating similar post-viral sequelae.<br><br>COVID-19 may reactivate latent EBV, especially in severe cases.<br><br>Shared symptoms include fatigue, brain fog, headaches, confusion, and myalgia.

		Prolonged EBV activity could lead to Hodgkin's lymphoma, peripheral B and T-cell lymphomas, and nasopharyngeal carcinoma	Complications may include hemolytic anemia, splenic rupture, hemophagocytic lymphohistiocytosis, and neurological problems.<br><br>EBV is associated with Burkitt's lymphoma.<br><br>EBV is linked to nasopharyngeal carcinoma.<br><br>EBV is connected to Hodgkin's lymphoma.<br><br>EBV can cause B-cell and T-cell lymphomas.<br><br>EBV is linked to oral hairy leukoplakia in immunocompromised individuals.<br><br>EBV can cause interstitial lymphocytic pneumonia.<br><br>EBV can lead to post-transplant lymphoproliferative disease.<br><br>EBV may contribute to mesenchymal tumors such as leiomyosarcoma.	Both viruses disrupt mitochondrial and immune pathways, contributing to long COVID symptoms.<br><br>EBV reactivation occurred in 4.5% of COVID-19 patients. EBV-positive patients had higher fever incidence, lower albumin, lower platelet count, and longer hospital stay.<br><br>Long COVID and EBV share symptoms like fatigue, cognitive dysfunction, and sleep disturbances.<br><br>EBV reactivation is proposed as a common mechanism, potentially inducing acquired immunodeficiency.<br><br>Both conditions show immune dysregulation, chronic inflammation, and viral reactivation, suggesting similar underlying pathophysiology.<br><br>COVID-19 patients showed a 2-fold higher EBV reactivation rate (27.1%) than non-COVID patients (12.5%).<br><br>EBV reactivation may contribute to COVID symptoms, severity, and long COVID, and was more common during the Omicron wave.<br><br>Shared symptoms and immune stress responses suggest possible overlap in post-viral complications.<br><br>The clinical presentation of these ulcers was similar to those caused by EBV, suggesting COVID-19 may trigger comparable immune responses or pathophysiological mechanisms.<br><br>A patient with mild COVID-19 developed worsening liver function, and tests confirmed EBV reactivation, which likely contributed to acute hepatitis.<br><br>EBV reactivation is frequently observed in long COVID and shares symptoms like fatigue, brain fog, sleep issues, myalgia, and GI upset.<br><br>EBV reactivation occurred in 65% of critically ill COVID-19 patients.<br><br>EBV/COVID-19 co-infection was associated with fever, higher CRP, and elevated AST, suggesting increased inflammation and potential disease severity.<br><br>EBV reactivation may serve as a marker of COVID-19 severity, possibly contributing to long COVID symptoms, inflammation, and immune suppression.

				EBV reactivation following SARS-CoV-2 infection is linked to increased inflammation, fatigue, and PASC (long COVID).<br><br>Both EBV and COVID-19 trigger NLRP3 inflammasome activation, contributing to persistent inflammation.<br><br>Those with EBV had higher rates of respiratory failure, ARDS, hypoproteinaemia, and elevated CRP and D-dimer, along with lower lymphocytes and albumin.<br><br>Shared symptoms included fatigue, myalgia, cognitive issues, fever, and pulmonary dysfunction.<br><br>EBV may contribute to long COVID via immune suppression, inflammation, and mitochondrial dysfunction, overlapping with autoimmune-like disorders.<br><br>Shared features include immune suppression, anemia, and hyperinflammation.<br><br>Both viruses showed symptom overlap including asthenia, sore throat, rash, anosmia, and ageusia.<br><br>Coinfection may cause diagnostic confusion and possibly aggravate clinical condition.<br><br>EBV reactivation is suggested as a potential contributor to long COVID symptoms like fatigue and neurocognitive dysfunction.<br><br>EBV and COVID-19 both increase the risk of periodontitis, possibly through immune modulation and inflammation.<br><br>EBV is associated with chronic and aggressive periodontitis, while COVID-19 may worsen periodontal disease and systemic outcomes via cytokine storms.<br><br>Suggested shared mechanisms include immune suppression and oral tissue vulnerability.<br><br>SARS-CoV-2 co-infection may trigger EBV lytic reactivation, leading to increased inflammation, fever, and immune suppression.<br><br>EBV reactivation has been linked to long COVID, and shared features include cytokine storms, CD8+ T cell loss, mitochondrial dysfunction, and upregulated ACE2 expression via EBV lytic genes.

				SARS-CoV-2 and EBV share pathogenic traits, including molecular mimicry, autoimmunity induction, and inflammation.<br><br>Both viruses are linked to multiple sclerosis, chronic fatigue, and post-COVID syndromes.<br><br>Authors suggest COVID-19 may trigger EBV reactivation, contributing to gastrointestinal inflammation, long COVID symptoms, and slow recovery.<br><br>Both viruses linked to immune dysregulation and chronic inflammatory responses.<br><br>Post-COVID and post-EBV share persistent fatigue as a primary symptom.
8	Pseudomonas aeruginosa	Increased biofilm production in microgravity, making it more resistant to treatments.<br><br>Causes opportunistic infections, especially in burn victims and immunocompromised individuals<br><br>P. aeruginosa demonstrated differential regulation of 167 genes and 28 proteins under spaceflight conditions<br><br>Increased production of virulence factors such as lecA, lecB, and rhlA, which are involved in bacterial adhesion, biofilm formation, and cytotoxicity<br><br>Spaceflight samples showed enhanced resistance to oxidative stress and biofilm formation, increasing the risk of persistent infections<br><br>Respiratory infections risk: P. aeruginosa has been found in astronaut microbiomes and is linked to lung infections in immunocompromised individuals<br><br>Impaired immune response under microgravity conditions led to increased bacterial load in blood.<br><br>Higher morbidity rates in irradiated and suspended mice due to reduced clearance of the infection	Causes infection and bacteremia mainly in immunocompromised individuals.<br><br>Commonly infects the lower respiratory tract, leading to pneumonia.<br><br>Can cause necrotizing bronchopneumonia in cystic fibrosis patients.<br><br>Frequently responsible for ventilator-associated pneumonia in hospitals.<br><br>May lead to endocarditis, osteomyelitis, urinary tract infections, gastrointestinal infections, and meningitis.<br><br>Can cause septicemia (blood poisoning).<br><br>Often causes corneal infections in contact lens wearers, leading to scarring or vision loss.<br><br>Infects burn wounds, potentially causing abscesses, sepsis, and tissue discoloration.<br><br>Associated with swimmer's ear (otitis externa).<br><br>Has a mortality rate of around 30%, which may be higher depending on health conditions.	Review not conducted due to time constraints.
9	Escherichia coli	Increased resistance to antibiotics.<br><br>Altered gene expression in microgravity, leading to enhanced survival and potential virulence<br><br>Increased antibiotic resistance observed in E. coli isolates from astronauts.<br><br>Spaceflight conditions led to changes in bacterial phenotype, increasing its resistance to colistin and kanamycin.	Causes abrupt onset of watery diarrhea without blood, pus, or mucus.<br><br>Diarrhea is usually mild to moderate but can lead to severe fluid loss.<br><br>Low-grade fever, nausea, and abdominal pain may occur.<br><br>Severe dehydration may develop, especially in neonates and children.	Review not conducted due to time constraints.

		Potential for opportunistic infections due to changes in intestinal flora	Illness typically lasts 2–5 days but can extend to 7 days or longer.<br><br>In severe cases, the disease can be life-threatening without treatment.<br><br>Estimated 800,000 deaths occur annually due to ETEC.<br><br>Most common in young children in developing countries and travelers from non-endemic regions.	
10	Staphylococcus aureus	S. aureus was detected in the upper respiratory tract of astronauts.<br><br>In-flight cross-contamination between astronauts was observed.<br><br>Increase in opportunistic infections due to changes in bacterial flora caused by spaceflight<br><br>Staphylococcus aureus was not detected in the astronaut's nasal microbiome before flight but was present after 3 months aboard the ISS, suggesting possible microbial colonization from the spacecraft environment<br><br>Increased relative abundance of Staphylococcus aureus in the nasal microbiome could contribute to upper respiratory symptoms such as congestion, rhinitis, and sneezing, which were commonly reported by astronauts	Causes food poisoning with symptoms like nausea, vomiting, abdominal pain, and diarrhea.<br><br>Leads to skin infections such as pimples, impetigo, folliculitis, blisters, and abscesses.<br><br>Can cause cellulitis, erythema, tenderness, and mild fever from animal bites.<br><br>Responsible for scalded skin syndrome, especially in neonates and children.<br><br>May lead to necrotizing fasciitis, a rare but life-threatening condition.<br><br>Produces toxins that can cause toxic shock syndrome (TSS), a severe illness with high fever, rash, hypotension, and multi-organ failure.<br><br>Can cause deep infections including endocarditis, peritonitis, necrotizing pneumonia, bacteremia, meningitis, osteomyelitis, and septic arthritis.	Review not conducted due to time constraints.
11	Klebsiella pneumoniae infections	Higher bacterial persistence in lung tissue under spaceflight-like conditions.<br><br>Increased pulmonary infection risk due to reduced granulocyte response	Causes nosocomial pneumonia, accounting for 7–14% of hospital-acquired cases.<br><br>Can lead to septicemia, urinary tract infections, and wound infections.<br><br>Commonly infects ICU patients and causes neonatal septicemias.<br><br>Responsible for community-acquired and hospital-acquired pneumonia with symptoms like fever, chills, and red currant jelly-like sputum.<br><br>May cause lung abscesses and necrosis of lung lobes.<br><br>Can lead to community-acquired meningitis and brain abscesses.<br><br>May cause pyogenic liver abscesses with symptoms like right upper quadrant pain, fever, and gastrointestinal discomfort.<br><br>Frequently causes urinary tract infections, including in patients with catheters.	Review not conducted due to time constraints.

			Can result in other infections such as endocarditis, peritonitis, necrotizing fasciitis, and septic arthritis.<br><br>Klebsiella granulomatis causes donovanosis, a chronic ulcerative disease of the genitalia.<br><br>Infections are more common in immunocompromised individuals, especially those with diabetes, alcoholism, or chronic illness.<br><br>Infections can be severe and associated with high morbidity and mortality.	
12	Staphylococcus epidermidis	Staphylococcus epidermidis was present throughout the mission but showed a shift in community composition	Pathogen Safety Data Sheet Not Available	Review not conducted due to time constraints.

Table S2: Comparison of guidelines for managing airborne infectious diseases in space and on Earth, including COVID-19. This table summarizes key recommendations from studies on airborne pathogens in space, standard Earth-based biosafety measures, and existing COVID-19 guidelines. It includes containment protocols, infection prevention strategies, immune system monitoring, microbial surveillance, and therapeutic considerations. Guidelines were also aligned using the Canadian Biosafety Handbook based on Containment Level 2 and Risk Group 2 classifications.

Guidelines for Airborne Infectious Diseases in Space	Guidelines for Airborne Infectious Diseases on Earth	Guidelines for COVID-19 on Earth
• Emphasis on the need for in-depth understanding of infection mechanisms in space<br>• Potential use of nutritional countermeasures and probiotics for infection prevention during spaceflight.<br>• Highlighting the need to monitor opportunistic pathogens in space environments, as increased virulence may pose risks to immunocompromised astronauts. Further research recommended.<br>• Emphasizes NASA's Health Stabilization Program (HSP) for infection prevention.<br>• Suggests vaccination protocols before space travel.<br>• Recommends in-flight isolation rooms and monitoring microbial changes during space missions.<br>• Recommends monitoring opportunistic pathogens in space environments.<br>• Suggests further research on microbial adaptability and infection risks for astronauts in microgravity conditions.<br>• Emphasizes the need for real-time microbial monitoring during space missions.<br>• Recommends biosensors for pathogen detection and antimicrobial resistance testing.<br>• Suggests evaluating astronaut immune function to mitigate infection risks in space.<br>• Recommends regular screening for microbial contamination before space travel.<br>• Suggests probiotic intake to maintain a healthy microbiome.<br>• Emphasizes the need for alternative therapies such as bacteriophage therapy and photodynamic therapy due to antibiotic resistance concerns in space.<br>• Suggests regular monitoring of P. aeruginosa in spacecraft water systems.<br>• Recommends biofilm disruption strategies to reduce infection risk in closed environments.<br>• Highlights the need for better antimicrobial resistance testing in space<br>• Recommends monitoring astronauts for viral reactivation pre- and post-flight.<br>• Suggests antiviral prophylaxis and immune-boosting interventions to prevent EBV-related complications in space.<br>• Calls for further research into radiation-induced viral reactivation mechanisms<br>• Monitoring and controlling airborne pathogens in spacecraft water and air systems.<br>• Developing countermeasures to prevent bacterial persistence in space habitats.<br>• Studying immune system responses further to mitigate infection risks during extended missions<br>• Monitoring for VZV reactivation in astronauts before, during, and after space missions.<br>• Suggests antiviral prophylaxis for astronauts with a history of VZV infection.<br>• Calls for further research into the long-term effects of VZV reactivation in space conditions<br>• Suggests continuous monitoring of Salmonella on spacecraft surfaces.<br>• Recommends studying astronaut microbiome shifts to assess infection risks in space.<br>• Calls for targeted countermeasures to reduce spaceflight-enhanced virulence<br>• Suggests continuous monitoring of Salmonella virulence factors in space environments.<br>• Recommends developing infection control strategies for long-duration missions.<br>• Calls for targeted antimicrobial measures to counteract spaceflight-induced bacterial virulence<br>• Continuous microbial monitoring of astronaut microbiomes before, during, and after spaceflight.<br>• Suggests further research on microbial transmission risks in space habitats.<br>• Calls for improved environmental controls to limit bacterial colonization in spacecraft	Based on All available Pathogen Safety Data Sheets, the pathogens studied in space are all classified as Containment Level 2 and Risk Group 2.<br><br>The Standard guidelines for these diseases according to Canadian Biosafety handbook are:<br><br>• Administrative controls (e.g., biosafety program management, training)<br>• Standardized procedures (e.g., work practices, personal protective equipment [PPE] use, and decontamination) that mitigate the risks associated with the activities conducted within the zone.<br>• Physical containment features include facility design (e.g., location of laboratory, surface finishes, access control)<br>• Provision of biosafety equipment, such as primary containment devices (e.g., biological safety cabinets [BSCs]) for certain activities.	Based on available Pathogen Safety Data Sheet on COVID-19, the pathogens studied in space are all classified as Containment Level 2 and Risk Group 2.<br><br>The Standard guidelines for these diseases according to Canadian Biosafety handbook are:<br><br>• Administrative controls (e.g., biosafety program management, training)<br>• Standardized procedures (e.g., work practices, personal protective equipment [PPE] use, and decontamination) that mitigate the risks associated with the activities conducted within the zone.<br>• Physical containment features include facility design (e.g., location of laboratory, surface finishes, access control)<br>• Provision of biosafety equipment, such as primary containment devices (e.g., biological safety cabinets [BSCs]) for certain activities.

Section and Topic	Item #	Checklist item	Location where item is reported
**TITLE**			
Title	1	Identify the report as a systematic review.	2-3
**ABSTRACT**			
Abstract	2	See the PRISMA 2020 for Abstracts checklist.	10-28
**INTRODUCTION**			
Rationale	3	Describe the rationale for the review in the context of existing knowledge.	32-148
Objectives	4	Provide an explicit statement of the objective(s) or question(s) the review addresses.	150-179
**METHODS**			
Eligibility criteria	5	Specify the inclusion and exclusion criteria for the review and how studies were grouped for the syntheses.	191-200; 231-236
Information sources	6	Specify all databases, registers, websites, organisations, reference lists and other sources searched or consulted to identify studies. Specify the date when each source was last searched or consulted.	182-189; 219-229
Search strategy	7	Present the full search strategies for all databases, registers and websites, including any filters and limits used.	182-189; 219-229
Selection process	8	Specify the methods used to decide whether a study met the inclusion criteria of the review, including how many reviewers screened each record and each report retrieved, whether they worked independently, and if applicable, details of automation tools used in the process.	191-200; 231-236
Data collection process	9	Specify the methods used to collect data from reports, including how many reviewers collected data from each report, whether they worked independently, any processes for obtaining or confirming data from study investigators, and if applicable, details of automation tools used in the process.	202-206; 238-241
Data items	10a	List and define all outcomes for which data were sought. Specify whether all results that were compatible with each outcome domain in each study were sought (e.g. for all measures, time points, analyses), and if not, the methods used to decide which results to collect.	247-264
	10b	List and define all other variables for which data were sought (e.g. participant and intervention characteristics, funding sources). Describe any assumptions made about any missing or unclear information.	266-285
Study risk of bias assessment	11	Specify the methods used to assess risk of bias in the included studies, including details of the tool(s) used, how many reviewers assessed each study and whether they worked independently, and if applicable, details of automation tools used in the process.	191-200; 231-236
Effect measures	12	Specify for each outcome the effect measure(s) (e.g. risk ratio, mean difference) used in the synthesis or presentation of results.	N/A
Synthesis methods	13a	Describe the processes used to decide which studies were eligible for each synthesis (e.g. tabulating the study intervention characteristics and comparing against the planned groups for each synthesis (item #5)).	191-200; 231-236
	13b	Describe any methods required to prepare the data for presentation or synthesis, such as handling of missing summary statistics, or data conversions.	202-206; 238-241
	13c	Describe any methods used to tabulate or visually display results of individual studies and syntheses.	205-206; 240-241; 250-252
	13d	Describe any methods used to synthesize results and provide a rationale for the choice(s). If meta-analysis was performed, describe the model(s), method(s) to identify the presence and extent of statistical heterogeneity, and software package(s) used.	N/A
	13e	Describe any methods used to explore possible causes of heterogeneity among study results (e.g. subgroup analysis, meta-regression).	N/A
	13f	Describe any sensitivity analyses conducted to assess robustness of the synthesized results.	N/A
Reporting bias assessment	14	Describe any methods used to assess risk of bias due to missing results in a synthesis (arising from reporting biases).	287-291
Certainty assessment	15	Describe any methods used to assess certainty (or confidence) in the body of evidence for an outcome.	N/A
**RESULTS**			
Study selection	16a	Describe the results of the search and selection process, from the number of records identified in the search to the number of studies included in the review, ideally using a flow diagram.	Figure 1 and 2
	16b	Cite studies that might appear to meet the inclusion criteria, but which were excluded, and explain why they were excluded.	300-303
Study characteristics	17	Cite each included study and present its characteristics.	489
Risk of bias in	18	Present assessments of risk of bias for each included study.	287-291

Section and Topic	Item #	Checklist item	Location where item is reported
studies			
Results of individual studies	19	For all outcomes, present, for each study: (a) summary statistics for each group (where appropriate) and (b) an effect estimate and its precision (e.g. confidence/credible interval), ideally using structured tables or plots.	Supplementary Table 1 and Table 2
Results of syntheses	20a	For each synthesis, briefly summarise the characteristics and risk of bias among contributing studies.	N/A
	20b	Present results of all statistical syntheses conducted. If meta-analysis was done, present for each the summary estimate and its precision (e.g. confidence/credible interval) and measures of statistical heterogeneity. If comparing groups, describe the direction of the effect.	N/A
	20c	Present results of all investigations of possible causes of heterogeneity among study results.	N/A
	20d	Present results of all sensitivity analyses conducted to assess the robustness of the synthesized results.	N/A
Reporting biases	21	Present assessments of risk of bias due to missing results (arising from reporting biases) for each synthesis assessed.	406-413
Certainty of evidence	22	Present assessments of certainty (or confidence) in the body of evidence for each outcome assessed.	N/A
**DISCUSSION**			
Discussion	23a	Provide a general interpretation of the results in the context of other evidence.	367-405
	23b	Discuss any limitations of the evidence included in the review.	406-413
	23c	Discuss any limitations of the review processes used.	414-422
	23d	Discuss implications of the results for practice, policy, and future research.	432-440
**OTHER INFORMATION**			
Registration and protocol	24a	Provide registration information for the review, including register name and registration number, or state that the review was not registered.	N/A
	24b	Indicate where the review protocol can be accessed, or state that a protocol was not prepared.	N/A
	24c	Describe and explain any amendments to information provided at registration or in the protocol.	N/A
Support	25	Describe sources of financial or non-financial support for the review, and the role of the funders or sponsors in the review.	485-486
Competing interests	26	Declare any competing interests of review authors.	499
Availability of data, code and other materials	27	Report which of the following are publicly available and where they can be found: template data collection forms; data extracted from included studies; data used for all analyses; analytic code; any other materials used in the review.	489

Table S3: PRISMA 2020 Checklist of this study.
